# Characterisation of the mechanical and fracture properties of a uni-weave carbon fibre/epoxy non-crimp fabric composite

**DOI:** 10.1016/j.dib.2016.01.010

**Published:** 2016-01-15

**Authors:** Thomas Bru, Peter Hellström, Renaud Gutkin, Dimitra Ramantani, Göran Peterson

**Affiliations:** a Swerea SICOMP, P.O. Box 104, 431 22 Mölndal, Sweden; b Division of Material and Computational Mechanics, Department of Applied Mechanics, Chalmers University of Technology, SE-412 96 Göteborg, Sweden; c Volvo Group Trucks Technology, Department 26547, AB2V, 405 08 Göteborg, Sweden

**Keywords:** Avg, average, CC, compact compression, CFRP, carbon fibre reinforced plastic, CNC, computer numerical control, CT, compact tension, CV, coefficient of variation, DCB, double cantilever beam, DIC, digital image correlation, ENF, end notched flexure, FRP, fibre reinforced plastic, FVF, fibre volume fraction, MMB, mixed-mode bending, NCF, non-crimp fabric, NL, nonlinearity method, Peak, maximum peak method, *R*-curve, crack resistance curves, RTM, resin transfer moulding, TT, through-the-thickness, VI, vacuum infusion, VO, visual observation method, Polymer matrix composite, Carbon fibre, Non-crimp fabric, Mechanical testing, Mechanical properties, Stress/strain curve, Fracture toughness

## Abstract

A complete database of the mechanical properties of an epoxy polymer reinforced with uni-weave carbon fibre non-crimp fabric (NCF) is established. In-plane and through-the-thickness tests were performed on unidirectional laminates under normal loading and shear loading. The response under cyclic shear loading was also measured. The material has been characterised in terms of stiffness, strength, and failure features for the different loading cases. The critical energy release rates associated with different failure modes in the material were measured from interlaminar and translaminar fracture toughness tests. The stress–strain data of the tensile, compressive, and shear test specimens are included. The load–deflection data for all fracture toughness tests are also included. The database can be used in the development and validation of analytical and numerical models of fibre reinforced plastics (FRPs), in particular FRPs with NCF reinforcements.

**Specifications Table**

**Table:** 

Subject area	*Composite materials*
More specific subject area	*Material characterisation/mechanics of composite materials*
Type of data	*Table and graphs, pictures*
How data was acquired	*Universal testing machines, strain gauges* (*Showa N22-FA-5-120-11-VS2 for the in-plane tensile tests, Kyowa KFG-3-120-C1-11L3M3R for the compressive tests and through-the-thickness tensile tests*)*, DIC system (ARAMIS 2M*(*-5M*) *from GOM GmbH*)*, travelling microscope*
Data format	*Raw data in CSV format and post-processed data in tables and graphs*
Experimental factors	*Mechanical and fracture properties a uni-weave NCF composite material*
Experimental features	*Stress/strain response, stiffness, strength, fracture toughness, failure features*
Data source location	*Sweden*
Data accessibility	*Data are included in this article*

## Value of the data

• This data set presents a complete mechanical characterisation of a CFRP system.
• The data can be used as input properties in analytical models.
• The data can be used as input parameters in finite element analyses and used for validation of results.
• The data can be compared to already available data for others CFRPs. The data can also be used in the development of future CFRPs, in particular those with NCF reinforcements.
• Guidelines for the mechanical and fracture characterisation of a given FRP material are provided.

## 1 Data

The stress–strain curves under the following loading cases are presented:

• in-plane longitudinal tension
• in-plane longitudinal compression
• in-plane transverse tension
• in-plane transverse compression
• through-the-thickness (TT) tension
• TT compression
• in-plane shear
• TT shear

The following terminology is used: 1-index refers to the longitudinal (to the fibre) direction in the reinforcement plane, 2-index refers to the transverse direction in the reinforcement plane, and 3-index refers to the TT direction w.r.t. the reinforcement plane. The stiffness and strength values are extracted from the stress–strain curves, and the specimen failure features reported.

Load–deflection curves are obtained from interlaminar fracture toughness tests in mode I, mode II and mixed-mode, and from translaminar fracture toughness tests. The energy release rates associated with the initiation of crack growth for the different tests are reported, as well as the crack resistance curves (*R*-curves).

The dimensions of the tests specimens are reported in Appendix A. The raw data for all test specimens are provided in CSV files in Appendix B.

## 2. Materials

**Fig. 1:**
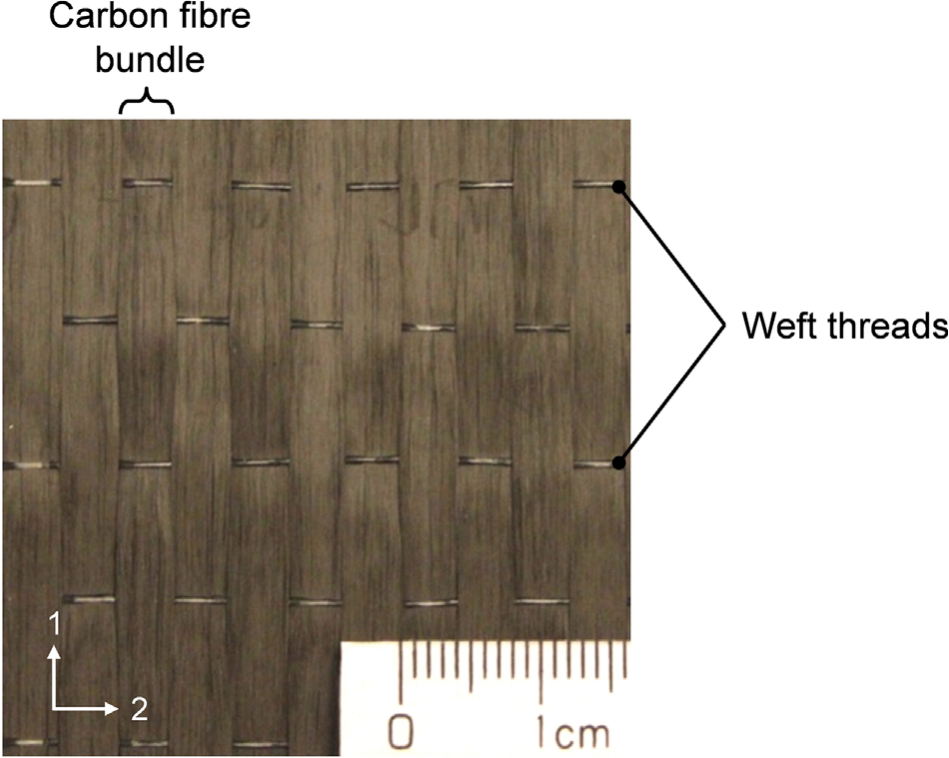
Photograph of the uni-weave NCF.

The carbon fibre reinforced plastic (CFRP) material system is an HTS45/LY556. The Hunstman LY556 epoxy resin was supplied by ABIC Kemi AB. The reinforcement layer is a 205 GSM uni-weave non-crimp fabric (NCF), from Porcher Industries. It consists of HTS45 E23 Tenax® carbon fibre bundles, which are held together by glass fibre/polyamide weft threads (Fig. 1). HTS45/LY556 laminates were manufactured by resin transfer moulding (RTM) and vacuum infusion (VI) processes, according to the epoxy resin manufacturer׳s recommendation. All the test specimens needed to build the data set were prepared from the laminates listed in Table 1. The fibre volume fraction (FVF) was estimated from the laminate thickness, the laminate layup, the area weight of the carbon fibres in the NCF, and the density of carbon fibres (data provided in [1], [2]).

## 3. Experimental design and methods

### 3.1 In-plane tensile and compressive properties

The test procedure for the tensile and compressive in-plane tests followed the ASTM standard D 3039 [3] and the ASTM standard D 3410 [4], respectively. Both longitudinal and transverse properties were measured. All specimens were tabbed with 1 mm thick glass fibre/epoxy laminates and equipped with strain gauges. The compressive specimens were initially polished to eliminate free edge effects.

**Fig. 2:**
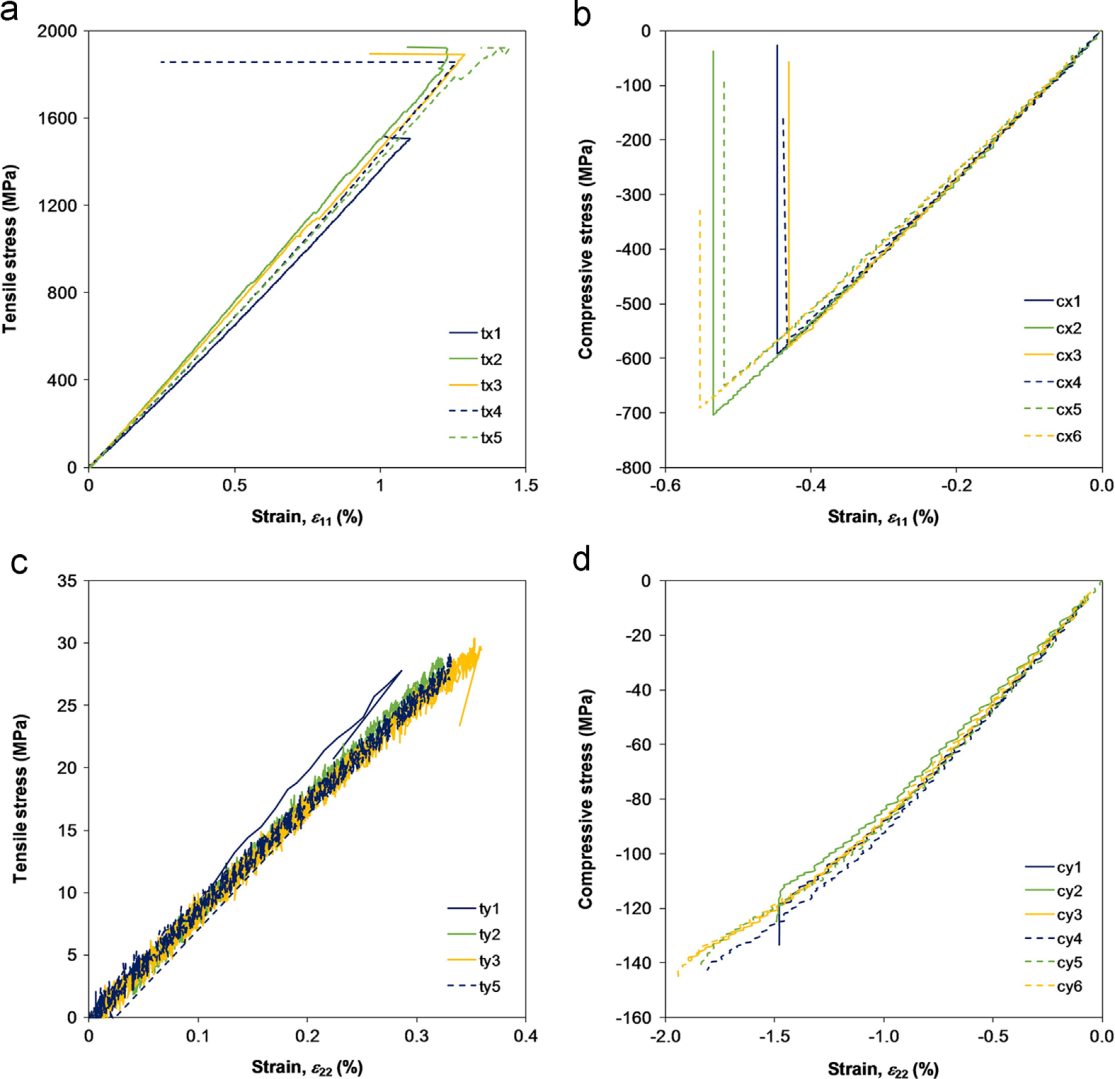
Stress–strain curves of the in-plane tensile and compressive tests; (a) longitudinal tension, (b) longitudinal compression, (c) transverse tension, and (d) transverse compression.

Table 2 and Fig. 2 report the results of the tests. The specimen bending in the gauge section, $B_{y}$, was evaluated in the compressive tests from the back-to-back strain measurements, according to the standard recommendation (Eq. 2 in [4]). Only the average between the two strain gauge readings was considered to construct the stress–strain curve. In the tensile tests, the strain transverse to the loading direction was also measured to evaluate the Poisson׳s ratios of the FRP material.

**Fig. 3:**
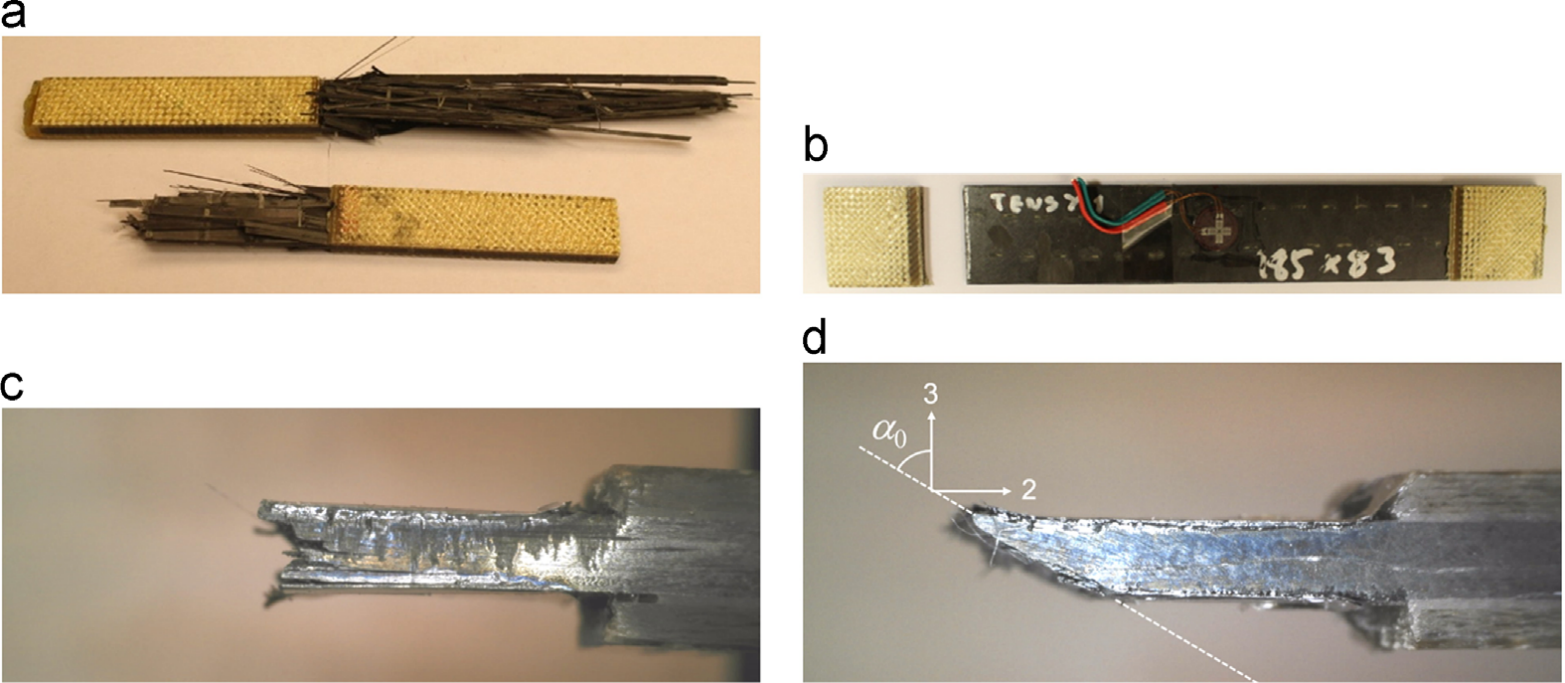
Specimen failures observed in in-plane tests; (a) longitudinal tension, (b) transverse tension, (c) longitudinal compression, and (d) transverse compression.

Longitudinal tensile specimens exhibited broom-like fracture, Fig. 3(a). Transverse tensile specimens failed in the gauge section at the end of the tabs, Fig. 3(b). Longitudinal compressive specimens failed by kink-band formation resulting in a stepped fracture surface, Fig. 3(c). Finally, transverse compressive specimens failed in a localised way with a smooth fracture surface oriented with an angle $\alpha_{0}$ to the direction transverse to the loading, Fig. 3(d).

### 3.2 Shear properties

Iosipescu tests, documented with the ASTM standard D 5379 [5], were performed to evaluate the material response under in-plane and TT shear (in the 1–3 plane) loading. The data was extracted from monotonic tests and cyclic tests. The latter consists of unloading/reloading cycles with an increasing level of applied load. The specimens were prepared with the fibres oriented along the specimen length. The specimens for in-plane shear testing were tabbed with a 1 mm thick glass fibre/epoxy laminate outside the notched region to increase their load bearing capacity. The material orthotropic ratios $\frac{E_{11}}{E_{22}}$ and $\frac{E_{11}}{E_{33}}$ were used to determine the opening angle of in-plane and TT shear specimens, according to the rescaling procedure proposed by Melin and Neumeister [6]. During the tests, the shear strain was determined by averaging strain measurements from the digital image correlation (DIC) system over a narrow band spanning the notch-to-notch axis of the specimen.

**Fig. 4:**
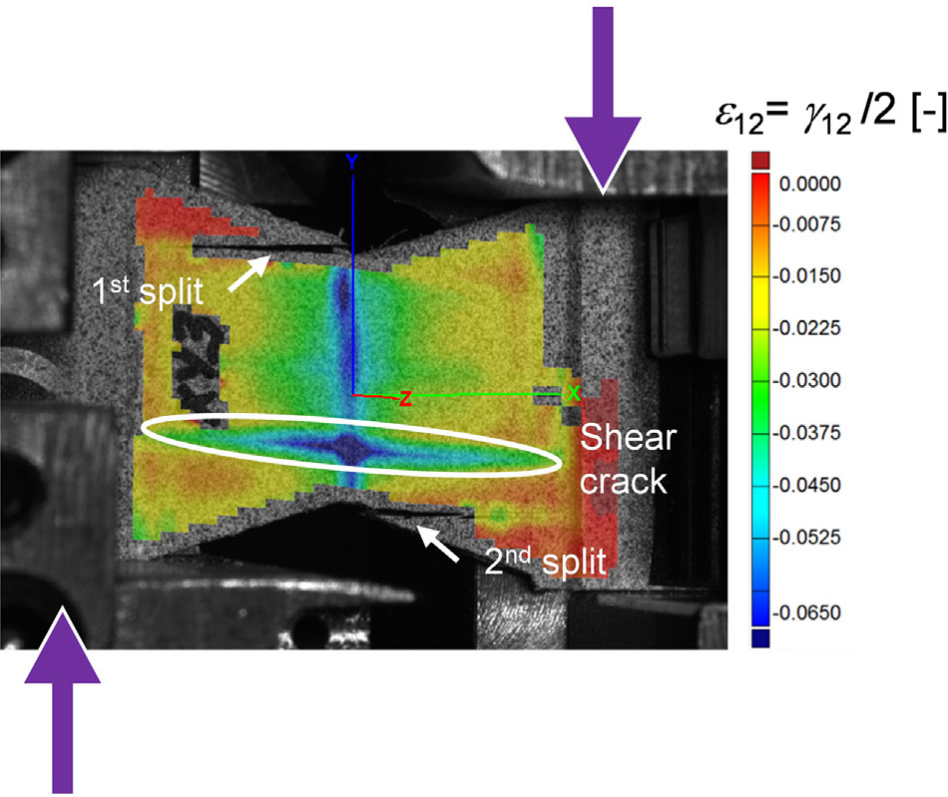
Failure of an in-plane Iosipescu specimen with the full-field strain measurements from the DIC system.

**Fig. 5:**
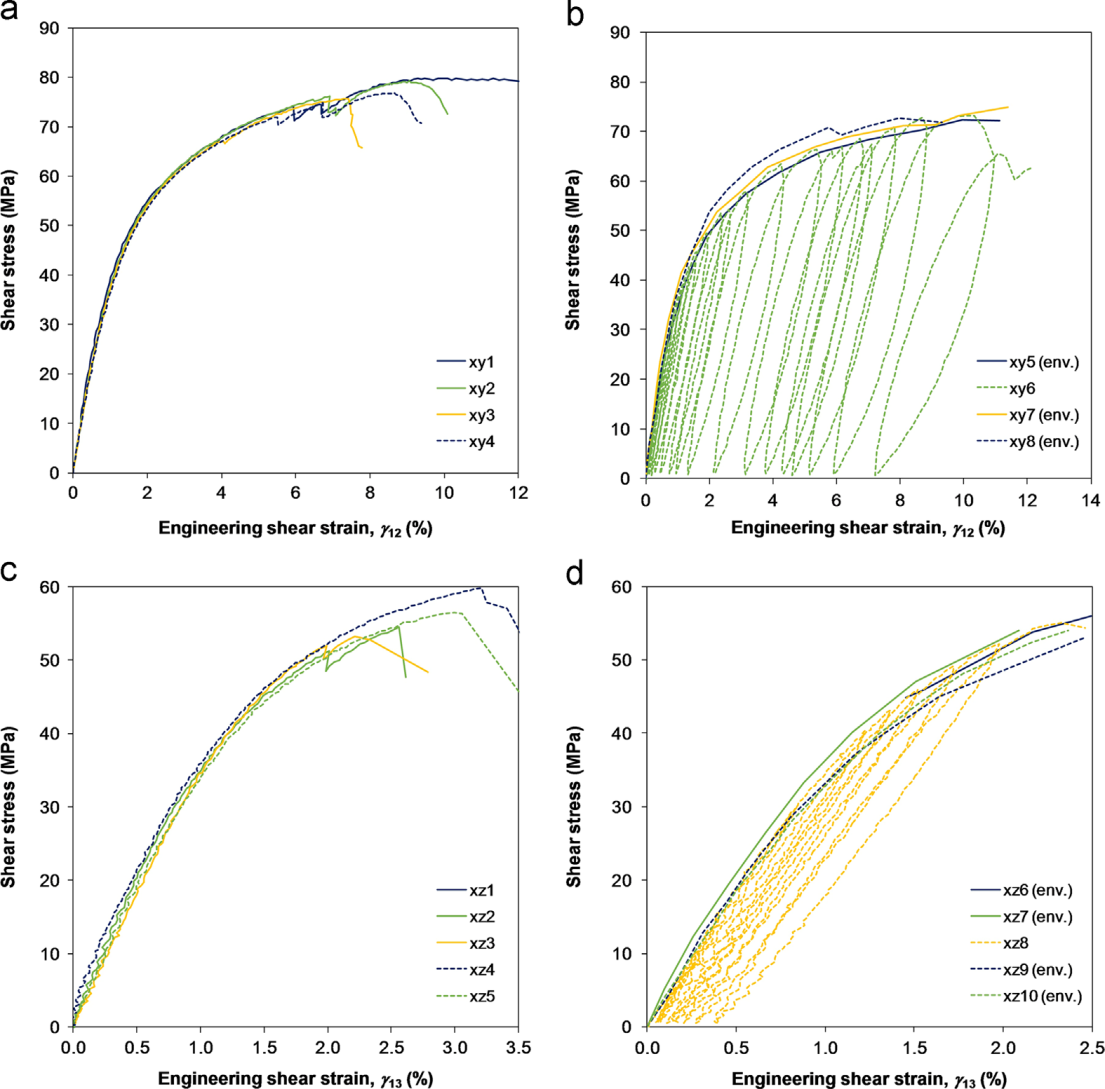
Stress–strain curves of the shear tests; (a) monotonic in-plane shear, (b) cyclic in-plane shear, (c) monotonic TT shear, and (d) cyclic TT shear. For the cyclic tests the entire response is shown for one specimen, and the envelopes of the stress–strain curves are shown for the other specimens.

The failure mode of the Iosipescu specimens was premature failure at the notches by splitting, followed by shear failure in the gauge section (Fig. 4). This failure mode is described as an acceptable failure mode in the test standard [5]. The shear data, reported in Table 3 and Fig. 5, indicate that the shear strength of the material is close to the splitting stress of the specimen. In some specimens shear failure occurred prior to splitting failure.

### 3.3 Interlaminar fracture toughness properties

Double cantilever beam (DCB), end notched flexure (ENF) and mixed-mode bending (MMB) interlaminar fracture toughness tests are documented by test method standards [7], [8], [9]. A mode mixity of 0.5 was chosen for the MMB tests, i.e. $G_{I}=G_{\mathrm{II}}$. For tests involving a mode I component, hinge caps were used instead of the standard piano hinges. In all test setups, the crack elongation was measured from the specimen edge with a travelling microscope.

**Fig. 6:**
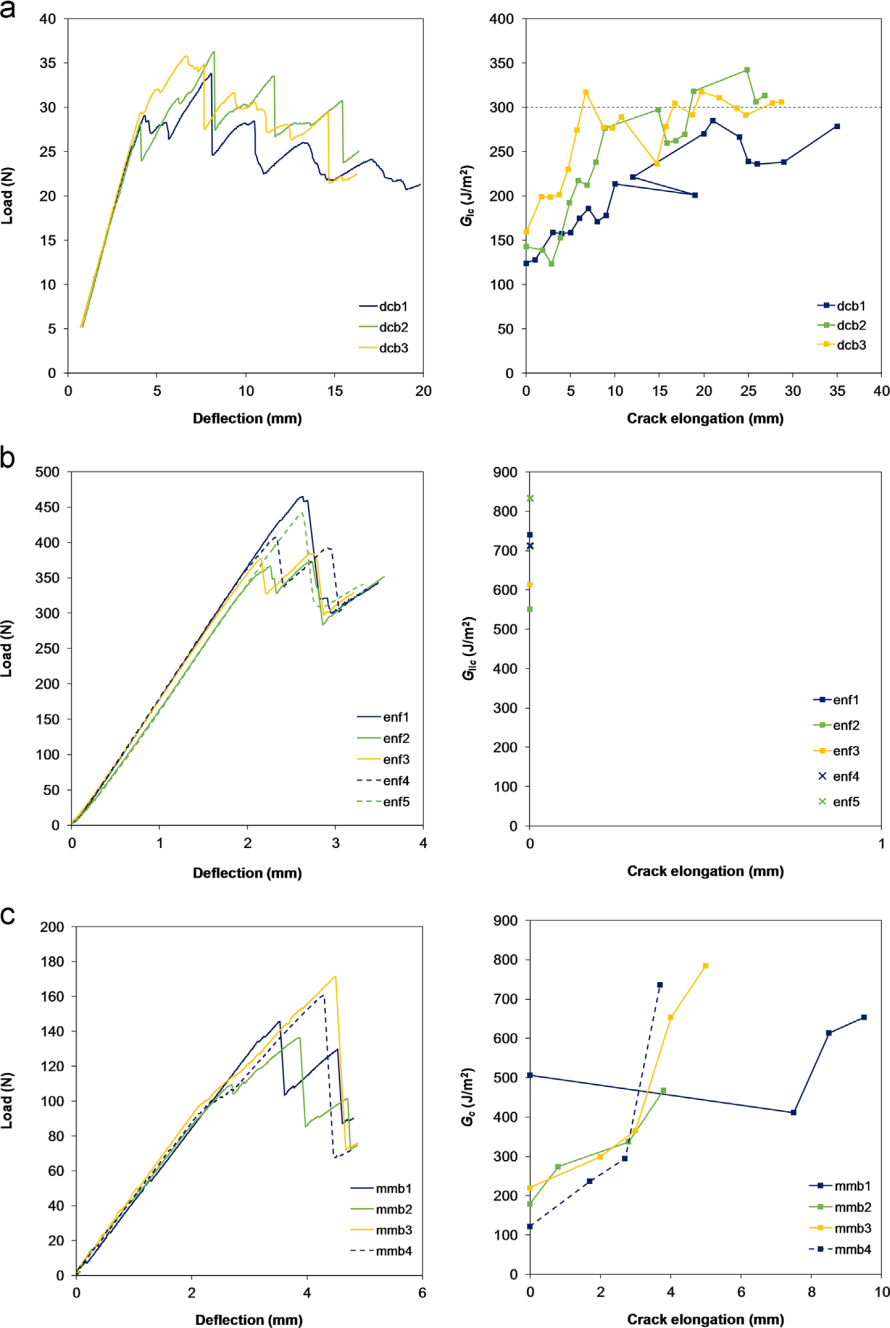
Load–deflection curves (left) and *R*-curves (right) obtained from (a) DCB tests, (b) ENF tests, and (c) MMB tests.

The critical energy release rates $G_{Ic}$ (mode I), $G_{\mathrm{II}c}$ (mode II), and $G_{c}$ (mixed-mode) were calculated following the procedure detailed in section 12.1.1 in [7], section 9.1 in [8], and section in 12.3.1 [9], respectively. From the load–deflection curves in Fig. 6, the initiation value of the critical energy release rates in each test was determined using the visual observation (VO), maximum peak (Peak), 5%/Max, and nonlinearity (NL) methods [7], [8], [9]. The critical energy release rate values at crack initiation for the different tests are reported in Table 4. The *R*-curves, in Fig. 6, were constructed using the VO method. For ENF tests, the crack generally made a single large jump as far as the loading point at the middle of the specimen, so no crack propagation value was measured. For the mode I tests, the *R*-curves in Fig. 6(a) are converging towards a propagation value of 300 J/m^2^.

**Fig. 7:**
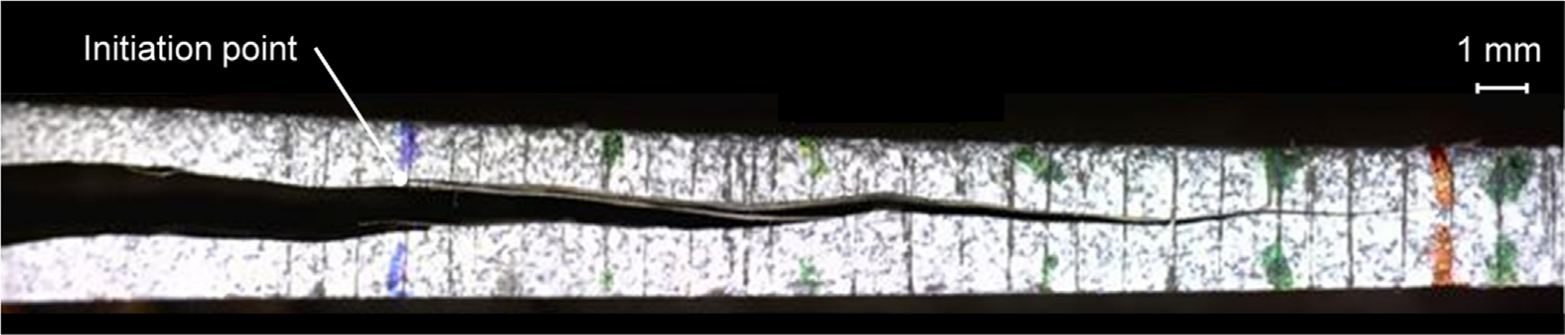
Crack path observed on a post-test MMB specimen. The initiation point indicates the end of the initial crack.

The fracture surfaces of DCB, ENF and MMB specimens were not perfectly flat but exhibited some waviness, which is specific of textile FRPs (Fig. 7). The formation of an undulating fracture surface is a toughness enhancing mechanism as it promotes slip-stick fracture processes.

### 3.4 TT tensile and compressive properties

**Fig. 8:**
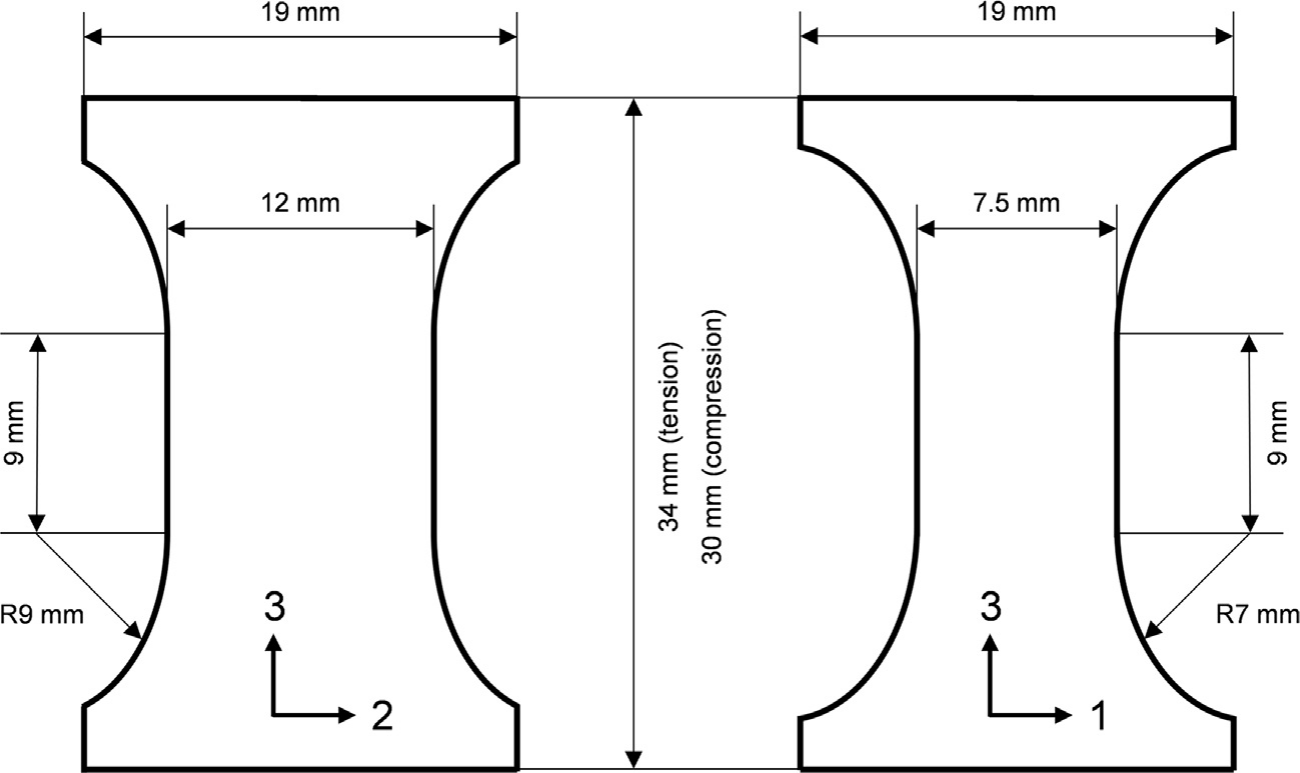
Dimensions of the double waisted specimens.

The TT tensile and compressive data were extracted using the double waisted specimen design proposed by Ferguson et al. [10]. A 1/2 scale version of the original specimen produces accurate data [10], but a 3/4 scale version was chosen to ensure that a sufficient amount of bundles of the NCF were present over the specimen gauge width (Fig. 8). The specimens were machined by a CNC milling machine using diamond-coated tools.

**Fig. 9:**
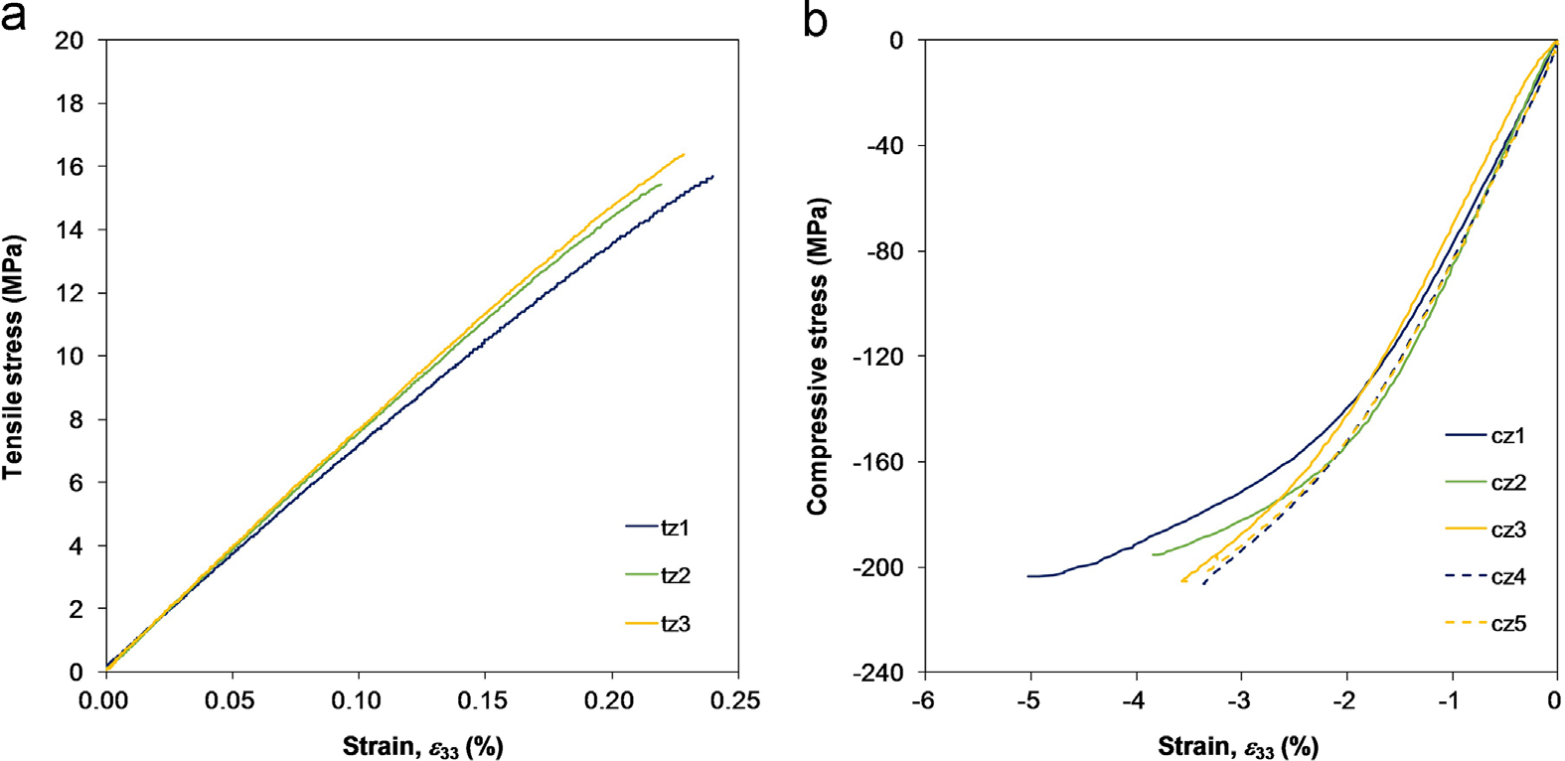
Stress–strain curves of the TT tensile (a) and compressive tests (b).

Table 5 reports the material data extracted from the stress-strain curves of the tensile and compressive tests (Fig. 9).

For the compressive tests, the specimens were simply loaded between two parallel platens in displacement control equivalent to an initial strain rate of approximately 2%/min. Back-to-back strain measurements and stereo DIC measurements indicated no specimen bending. The strains were averaged from the DIC measurements over the entire surface of constant gauge section. The surface monitored by the DIC system was not always the same in all specimens so that the evaluation of both Poisson׳s ratios $\nu_{32}$ and $\nu_{31}$ was possible.

For the tensile loading configuration, rod end bearings were attached to the universal testing machine to prevent the introduction of moments in the specimens. The specimen end surfaces were adhesively bonded to two steel plates connected to the bearings. Strain gauges were bonded at the centre of the wider surfaces of the specimen, and the average of the two strain readings was considered to construct the stress–strain curves. In two specimens, the strain gauges produced inaccurate signals and the strain data were discarded. However, the strength values associated with these two specimens are considered reliable.

**Fig. 10:**
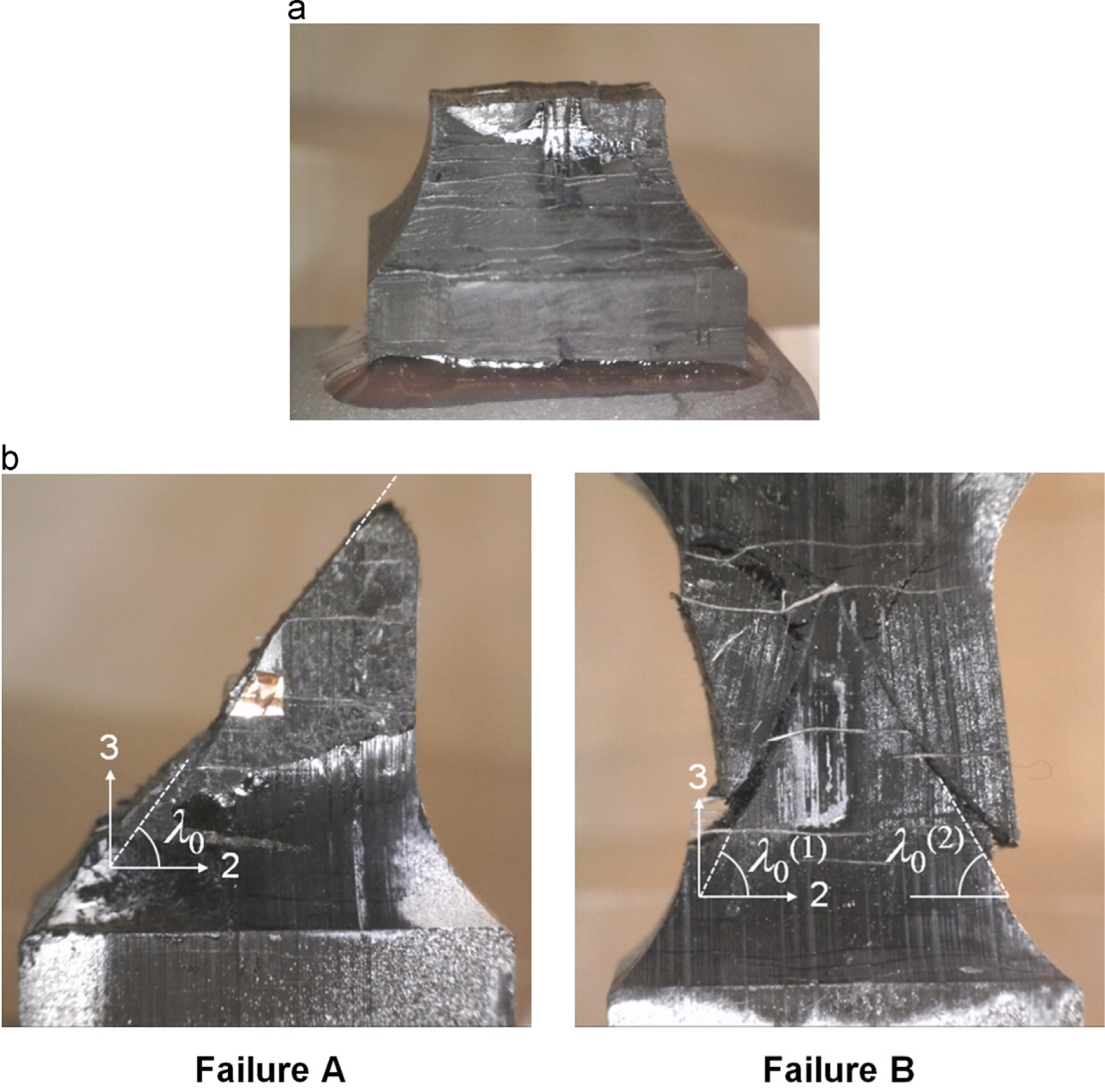
Failure of the double waisted specimens; in tension (a), and in compression (b).

Fig. 10 shows the different specimen failure modes observed during testing. The adhesive bond remained intact in all tensile specimens, which fractured in a region close to the waist radius (Fig. 10 (a)). Two failure modes were observed in the compressive case, Fig. 10 (b), and a fracture angle, $\lambda_{0}$, was defined.

### 3.5 Translaminar fracture toughness properties

**Fig. 11:**
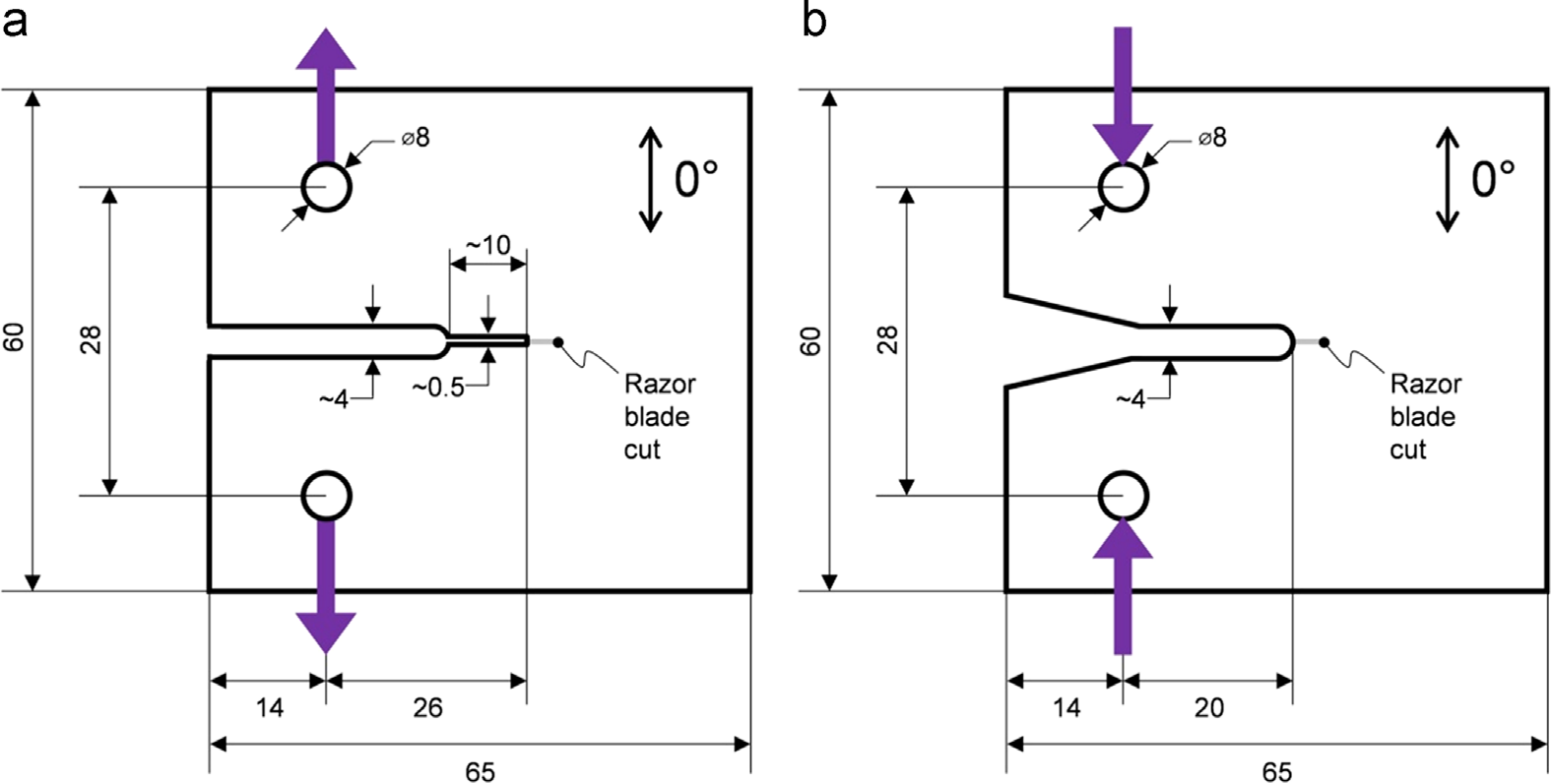
Dimensions of the CT specimens (a) and CC specimens (b); in mm.

The test procedure described by Pinho et al. [11] was followed to determine the energy associated with fibre breakage in tension and in compression, using compact tension (CT) and compact compression (CC) specimens, respectively. Fig. 11 shows the geometry of the specimens. The machining of the notches was as follows: first a circular saw was used to make a wide cut, then a 0.5 mm wide notch was achieved using a precision low-speed saw (only for CT specimens), and finally a razor blade was used to create a sharp pre-crack. During testing, the load was introduced using steel cylinders through the holes of the CT/CC specimen.

Cross-ply specimens are needed to prevent splitting at the notch when the crack initiates. The data reduction scheme, based on Eqs. (1)(2), (3), was followed to extract the critical energy release rate for the 0°-plies in tension and in compression. In Eq. (1), the critical energy release rate for the laminate is calculated from the measurement of the critical load $P_{c}$ at crack initiation. t is the thickness of each specimen. The unit energy release rate $G_{I|\mathrm{unit}}$ is found by calculating the *J*-integral of the specimen configuration (geometry and layup considered) with finite element methods.

(1) $$G_{Ic\mathrm{|lam}}=\frac{G_{\mathrm{I|unit}}P_{c}^{2}}{t^{2}}$$

From the critical energy release rate for the laminate, the critical energy release rate for the 0°-plies is found using Eqs. (2), (3), respectively,

(2) $$G_{Ic|0{}^{\circ}\mathrm{tensile}}=\frac{t}{t_{0{}^{\circ}}}G_{Ic\mathrm{|lam tensile}}-\frac{t_{90{}^{\circ}}}{t_{0{}^{\circ}}}G_{Ic,\mathrm{in}}$$

(3) $$G_{Ic|0{}^{\circ}\mathrm{compressive}}=\frac{t}{t_{0{}^{\circ}}}G_{Ic\mathrm{|lam compressive}}-\frac{{\sqrt{2}t}_{90{}^{\circ}}}{t_{0{}^{\circ}}}G_{\mathrm{II}c,\mathrm{in}}$$

where $t_{0{}^{\circ}}$ is the total thickness of the 0°-plies, and $t_{90{}^{\circ}}$ the total thickness of 90°-plies. The values for $G_{Ic,\mathrm{in}}$ and $G_{\mathrm{II}c,\mathrm{in}}$ were taken from in Table 4. The results from the data reduction scheme are presented in Table 6.

## Figures and Tables

**Table 1:** Plate specifications.

**Plate**	**Layup**		**Thickness (mm)**	**FVF (%)**	**Manufacturing process**	**Cure+post-cure**	**Cure pressure (bar)**
UD1	[0]_10_		1.83	61	RTM	4 h 80 °C+4 h 140 °C	3
UD2	[0]_187_		35/38	55/60^a^	VI	4 h 80 °C+4 h 140 °C	0.5
UD3^b^	[0]_16_		3.04	59	RTM	18 h 80 °C+4 h 140 °C	3
CP1		[0/90]_5s_	4.05	55	RTM	18h 80 °C+4 h 140 °C	3

a Considering 35 and 38 mm for the laminate thickness.

b 7.5 micron polyimide film insert in the midplane of the laminate.

**Table 2:** In-plane tensile/compressive properties.

**Specimen**	**Modulus**	**Poisson ratio**	**Strength**	**Strain at failure**	**Fracture angle**^a^	**Bending, By (%)**			
Transverse	$E_{22c}$ (GPa)		$Y_{c}$ (MPa)	$\varepsilon_{22\mathrm{cu}}$ (%)	$\alpha_{0}$ (deg)	(0.2%ε)		($\varepsilon_{22\mathrm{cu}}$)	
compression	(0–0.3%ε)					

cy1	9.4		118	1.48	65	−			−
cy2	8.5		114	1.47	53	2.2			-1.5
cy3	9.2		139	1.89	70	-0.5			2.4
cy4	9.7		140	1.79	64	2.5			7.5
cy5	9.7		133	1.78	56	3.5			8.5
cy6	9.0		138	1.88	65	5.6			3.8
*Avg.* (*CV*)	*9.3* (*5%*)		*130* (*9%*)	*1.71* (*11%*)	*62* (*10%*)				

Longitudinal	$E_{11c}$ (GPa)		$X_{c}$ (MPa)	$\varepsilon_{11\mathrm{cu}}$ (%)		(0.2%ε)		($\varepsilon_{11\mathrm{cu}}$)	
compression	(0.1–0.2%ε)						

cx1	134		591	0.45		3.8			3.6
cx2	137		703	0.53		6.4			14.0
cx3	135		579	0.43		-6.8			6.5
cx4	129		572	0.43		3.8			1.8
cx5	127		649	0.52		4.6			11.4
cx6	130		690	0.55		-26.2			29.5
*Avg. (CV)*	*132* (*3%*)		*631* (*9%*)	*0.49* (*11%*)					

Transverse	$E_{22t}$ (GPa)	$v_{21}$ (–)	$Y_{t}$ (MPa)	$\varepsilon_{22\mathrm{tu}}$ (%)					
tension	(0.05–0.2%ε)	(0.05–0.2%ε)					
ty1	9.6	0.032	27.8	0.29					
ty2	9.6	0.027	28.8	0.32					
ty3	7.8	–	30.3	0.36					
ty4	–b	–b	29.3	–b					
ty5	8.8	–	29.7	0.33					
*Avg.* (*CV*)	*9.0* (*10%*)	*0.029* (*12%*)	*29.2* (*3%*)	*0.32* (*9%*)					

Longitudinal	$E_{11t}$ (GPa)	$v_{12}$ (–)	$X_{t}$ (MPa)	$\varepsilon_{11\mathrm{tu}}$ (%)					
tension	(0.1–0.3%ε)	(0.1–0.3%ε)					
tx1	129	0.23	1506	1.10					
tx2	152	0.34	1889	1.23					
tx3	146	0.25	1891	1.29					
tx4	136	0.27	1851	1.25					
tx5	137	0.33	1796	1.26					
*Avg.* (*CV*)	*140* (*6%*)	*0.28* (*17%*)	*1787* (*9%*)	*1.23* (*6%*)					

a Defined in Fig. 3(d).

b No strain reading.

**Table 3:** In-plane shear and TT shear properties.

**Test/specimen**	**Modulus**	**Strength**	**Strain at failure**	**Shear stress at splitting**	**Shear strain at splitting**
In-plane shear	$G_{12}$ (GPa)	$S_{12}$ (MPa)	$\gamma_{12u}$ (%)	(MPa)	(%)
(monotonic)	(0.2–0.4%γ)				

xy1	4.8	79.8	11.3	74.1^a^	5.9^a^
xy2	4.5	79.0	9.2	76.2^a^	6.9^a^
xy3	4.1	75.7	7.4	75.7^a^	7.4^a^
xy4	4.2	76.8	8.7	72.0^a^	5.5^a^
*Avg.* (*CV*)	*4.4* (*7%*)	*77.8* (*3%*)	*9.1* (*18%)*	*74.5* (*3%*)	*6.4* (*14%*)

In-plane shear	$G_{12}$ (GPa)	$S_{12}$ (MPa)	$\gamma_{12u}$ (%)	(MPa)	(%)
(cyclic)	(0.2–0.4%γ)				

xy5	4.2	72.2	11.1	68.5^a^	7.0^a^
xy6	4.5	73.3	10.1	66.1^a^	5.8^a^
xy7	4.2	74.8	11.4	69.0^a^	6.4^a^
xy8	4.3	71.8	9.3	69.3^a^	6.1^a^
*Avg.* (*CV*)	*4.3* (*3%*)	*73.0* (*2%*)	*10.5* (*9%*)	*68.2* (*2%*)	*6.3* (*8%*)

TT shear	$G_{13}$ (GPa)	$S_{13}$ (MPa)	$\gamma_{13u}$ (%)	(MPa)	(%)
(monotonic)	(0.2–0.4%γ)				

xz1	3.8	59.4	3.4	59.3^a^	3.2^a^
xz2	3.9	54.5	2.6	51.2^a^	2.0^a^
xz3	3.5	53.3	2.2	52.0^a^	2.0^a^
xz4	3.4	59.8	3.2	59.8	3.2
xz5	3.9	56.4	3.0	56.4	3.0
*Avg.* (*CV*)	*3.7* (*6%*)	*56.7* (*5%*)	*2.9* (*17%*)	*55.7* (*7%*)	*2.7* (*24%*)

TT shear	$G_{13}$ (GPa)	$S_{13}$ (MPa)	$\gamma_{13u}$ (%)	(MPa)	(%)
(cyclic)	(0.2–0.4%γ)				

xz6	−b	56.0	2.5	42.5^a^	1.4^a^
xz7	3.9	50.4	2.1	−	−
xz8	3.7	55.0	2.3	−	−
xz9	4.0	53.0	2.5	53.0	2.5
xz10	3.5	54.1	2.4	54.1	2.4
*Avg.* (*CV*)	*3.8* (*6%*)	*53.7* (*4%*)	*2.3* (*7%*)	*49.8* (*13%*)	*2.1* (*29%*)

a Stress and strain levels associated to the first split.

b No load measurement in the range of modulus calculations.

**Table 4:** Initiation values of the critical energy release rates from the interlaminar fracture toughness tests.

**Test/specimen**	**Initiation value for the critical energy release rate (J/m^2^)**			
DCB (mode I)	VO		5%/Max	NL

dcb1	144		147	143
dcb2	143		143	137
dcb3	160		165	153
*Avg.* (*CV*)	*149* (*6%*)		*152* (*8%*)	*144* (*6%*)

ENF (mode II)	VO	Peak		

enf1	740	900		
enf2	551	607		
enf3	613	614		
enf4	713	721		
enf5	834	854		
*Avg.* (*CV*)	*690* (*16%*)	*739* (*18%*)		

MMB (mixed-mode)	VO	Peak	5%/Max	NL

mmb1	507	510	491	432
mmb2	179	476	304	304
mmb3	220	662	285	221
mmb4	122	603	246	199
*Avg.* (*CV*)	174*/257 (*28/67%*)	*563* (*15%*)	*332* (*33%*)	*289* (*37%*)

* Excluding deviant value of 507 for specimen. A possible explanation for the high toughness measured for specimen mmb1 is the presence of a rather uneven crack surface observed just at the location of crack initiation. The high energy built up at this location is finally released once a sufficient load is achieved, resulting in an instantaneous crack growth over 8 mm (see R-curve in Fig. 6(c)).

**Table 5:** TT tensile/compressive properties.

**Test/Specimen**	**Modulus**	**Poisson ratio**		**Strength**	**Strain at failure**	**Failure angle**
Compression	$E_{33c}$ (GPa)	$v_{32}$ (–)	$v_{31}$ (–)	$Z_{c}$ (MPa)	$\varepsilon_{33\mathrm{cu}}$ (%)	$\lambda_{0}$ (deg)
	(0.4–0.7%ε)	(0.4–0.7%*ε*)	(0.4–0.7%ε)			
cz1	7.7	0.43		204	5.03	56^a^
cz2	9.0	0.43		195	3.85	53^b^
cz3	7.9		0.02	206	3.50	54^b^
cz4	8.0		0.02	206	3.36	56^a^
cz5	7.9		0.02	203	3.34	52^a^
*Avg.* (*CV*)	*8.1* (*6%*)	*0.43* (*0%*)	*0.02* (*0%*)	*203* (*2%*)	*3.81* (*19%*)	*54* (*4%*)

Tension	$E_{33t}$ (GPa)			$Z_{t}$ (MPa)	$\varepsilon_{33\mathrm{tu}}$ (%)	
(0.01-0.05%ε)			
tz1	7.1			15.7	0.24	
tz2	7.1			15.4	0.22	
tz3	7.8			16.4	0.23	
tz4	−c			13.1	−c	
tz5	−c			13.0	−c	
*Avg.* (*CV*)	*7.3* (*5%*)			*14.7* (*11%*)	*0.23* (*5%*)	

a Failure mode B, according to Fig. 10(b). The average of the two fracture plane angles is used.

b Failure mode A, according to Fig. 10(b).

c No strain reading.

**Table 6:** Initiation values of the critical energy release rates from the translaminar fracture toughness tests.

**Test/Specimen**	**Initiation value for the critical energy release rate (kJ/m**^**2**^**)**	
Compact compression	$G_{I\mathrm{c|}\mathrm{lam}\mathrm{compressive}}$	$G_{I\mathrm{c|}0{}^{\circ}\mathrm{compressive}}$

cc1	53.7	107.1
cc2	49.8	99.2
*Avg.* (*CV*)	*51.8* (*5%*)	*103.1* (*5%*)

Compact tension	$G_{I\mathrm{c|}\mathrm{lam}\mathrm{tensile}}$	$G_{I\mathrm{c|}0{}^{\circ}\mathrm{tensile}}$

ct1	32.3	64.1
ct2	35.2	70.0
*Avg.* (*CV*)	*33.7* (*6%*)	*67.1* (*6%*)

**Table 7:** Information on the test specimens.

**Specimen**	**Plate**	**Thickness (mm)**	**Width (mm)**	**Gauge length(mm)**	**Comments**
Transverse compression					
cy1	UD1	1.88	9.71	10.29	One strain gauge
cy2	UD1	1.93	9.77	10.70	−
cy3	UD1	1.93	9.78	10.89	−
cy4	UD1	1.94	9.87	10.46	−
cy5	UD1	1.92	9.81	10.74	−
cy6	UD1	1.95	9.72	10.45	−
Longitudinal compression					
cx1	UD1	1.75	9.79	10.15	−
cx2	UD1	1.75	9.81	10.21	−
cx3	UD1	1.78	9.90	10.17	−
cx4	UD1	1.78	9.91	10.16	−
cx5	UD1	1.79	9.86	10.20	−
cx6	UD1	1.79	10.00	10.22	−
Transverse tension					
ty1	UD1	1.80	25.00	125	−
ty2	UD1	1.80	25.00	125	−
ty3	UD1	1.83	14.95	-	One strain gauge
ty4	UD1	1.81	24.80	124	No strain gauge
ty5	UD1	1.87	24.20	122	One strain gauge
Longitudinal tension					
tx1	UD1	1.80	11.99	90	−
tx2	UD1	1.80	12.02	90	−
tx3	UD1	1.81	12.02	90	−
tx4	UD1	1.80	12.04	90	−
tx5	UD1	1.80	11.96	86	−


**Specimen**	**Plate**	**Thickness (mm)**	**Gauge length (mm)**	**Notch angle(°)**	**Comments**
In-plane shear (monotonic)					
xy1	UD1	1.85	12.11	141	–
xy2	UD1	1.76	12.14	141	–
xy3	UD1	1.80	12.17	141	–
xy4	UD1	1.79	12.16	141	–
In-plane shear (cyclic)					
xy5	UD1	1.87	12.23	141	20 cycles
xy6	UD1	1.85	12.24	141	24 cycles
xy7	UD1	1.85	12.17	141	21 cycles
xy8	UD1	1.85	12.19	141	21 cycles
TT shear (monotonic)					
xz1	UD2	4.19	11.38	142	–
xz2	UD2	4.17	11.38	142	–
xz3	UD2	4.07	11.32	142	–
xz4	UD2	3.91	10.57	142	–
xz5	UD2	4.17	11.30	142	–
TT shear (cyclic)					
xz6	UD2	4.27	11.32	142	15 cycles ^(1)^
xz7	UD2	4.31	11.32	142	10 cycles
xz8	UD2	4.11	11.23	142	11 cycles
xz9	UD2	4.20	11.34	142	12 cycles
xz10	UD2	4.04	11.25	142	12 cycles
^(1)^*Only the last 4 cycles recorded.*					

**Specimen**	**Plate**	**Initial crack length**^**(1)**^**(mm)**	**Thickness (mm)**	**Width (mm)**	**Length (mm)**
DCB (mode I)					
dcb1	UD3	48.9	3.05	19.72	Approx. 180
dcb2	UD3	48.6	3.04	19.64	Approx. 180
dcb3	UD3	48.8	3.03	19.67	Approx. 180
ENF (mode II)					
enf1	UD3	35	3.04	19.74	Approx. 180
enf2	UD3	35	3.05	19.75	Approx. 180
enf3	UD3	36	3.06	19.73	Approx. 180
enf4	UD3	36	3.02	19.73	Approx. 180
enf5	UD3	35	3.04	19.73	Approx. 180
MMB (mixed-mode)					
mmb1	UD3	28.8	3.03	19.71	Approx. 160
mmb2	UD3	28.5	3.02	19.72	Approx. 160
mmb3	UD3	27.4	3.03	19.68	Approx. 160
mmb4	UD3	27.6	3.02	19.71	Approx. 160
^(1)^*Measured after testing by opening completely each specimen*.					

**Specimen**	**Plate**	**Height (mm)**	**Gauge section (mm x mm)**	**Comments**
Compression				
cz1	UD2	30.01	7.49×11.88	−
cz2	UD2	30.00	12.14×7.49	Fibres running along the widest surface
cz3	UD2	30.03	7.52×12.10	−
cz4	UD2	30.03	7.54×12.00	−
cz5	UD2	30.03	7.54×11.97	−
Tension				
tz1	UD2	34.11	7.64×11.98	−
tz2	UD2	32.02	7.66×11.87	−
tz3	UD2	34.04	7.64×11.74	−
tz4	UD2	34.04	7.53×12.02	−
tz5	UD2	34.02	7.57×12.02	−


**Specimen**	**Plate**	**Initial crack length (mm)**	**Thickness (mm)**	**Width (mm)**	**Height (mm)**
Compact compression					
cc1	CP1	20.18	4.09	65.19	60.04
cc2	CP1	20.33	4.03	65.15	59.96
Compact tension					
ct1	CP1	26.96	4.05	65.12	60.03
ct2	CP1	26.61	4.05	65.15	60.30
